# Disentangling the influence of earthworms in sugarcane rhizosphere

**DOI:** 10.1038/srep38923

**Published:** 2016-12-15

**Authors:** Lucas P. P. Braga, Caio A. Yoshiura, Clovis D. Borges, Marcus A. Horn, George G. Brown, Harold L. Drake, Siu M. Tsai

**Affiliations:** 1 Cell and Molecular Biology Laboratory, Center for Nuclear Energy in Agriculture (CENA), University of Sao Paulo (USP), Piracicaba, Brazil; 2 Institute of Microbiology, Leibniz University Hannover, Hannover, Germany; 3 Department of Ecological Microbiology, University of Bayreuth, Germany; 4 Embrapa Florestas, Colombo, Brazil

## Abstract

For the last 150 years many studies have shown the importance of earthworms for plant growth, but the exact mechanisms involved in the process are still poorly understood. Many important functions required for plant growth can be performed by soil microbes in the rhizosphere. To investigate earthworm influence on the rhizosphere microbial community, we performed a macrocosm experiment with and without *Pontoscolex corethrurus* (EW+ and EW−, respectively) and followed various soil and rhizosphere processes for 217 days with sugarcane. In EW+ treatments, N_2_O concentrations belowground (15 cm depth) and relative abundances of nitrous oxide genes (*nos*Z) were higher in bulk soil and rhizosphere, suggesting that soil microbes were able to consume earthworm-induced N_2_O. Shotgun sequencing (total DNA) revealed that around 70 microbial functions in bulk soil and rhizosphere differed between EW+ and EW− treatments. Overall, genes indicative of biosynthetic pathways and cell proliferation processes were enriched in EW+ treatments, suggesting a positive influence of worms. In EW+ rhizosphere, functions associated with plant-microbe symbiosis were enriched relative to EW− rhizosphere. Ecological networks inferred from the datasets revealed decreased niche diversification and increased keystone functions as an earthworm-derived effect. Plant biomass was improved in EW+ and worm population proliferated.

Earthworms have a great ability to modify their habitats. These animals are recognized as ecosystem engineers due to their ability to convert soils into specialized functional domains, such as the drilosphere (consisting of casts, burrows and the worms themselves)^1^ that can regulate soil nutrient fluxes well beyond the life-span of an individual earthworm^2^. Therefore, earthworms can improve plant growth by enhancing organic matter mineralization and improving soil porosity and water content^3^,^4^,^5^,^6^,^7^,^8^. However, the determination of the particular mechanisms connecting the promotion of beneficial soil functions and plant growth is more complex due to multiple interactions among the factors involved. For example, nitrogen is an essential nutrient for plant growth and its availability is limited in most terrestrial ecosystems^9^. A meta-analysis recently suggested that the benefits of earthworms would arise mainly from its capacity to improve the release of nitrogen trapped in organic matter^10^. Nevertheless, enhanced nitrogen release does not always explain plant growth in the presence of earthworms^11^. Blouin *et al*.^11^ tested the effect of earthworms on plant biomass over N-gradient conditions, and demonstrated that the beneficial effect on biomass improvement was independent of the variations in N concentrations. In their study, the hypothesis that the main effect of earthworms on plant production is due to increased N mineralization was rejected, suggesting a more complex mechanism in which not only mineralization of nutrients but also plant growth regulators are involved in the process by which earthworms improve plant biomass^11^,^12^. Such compounds have already been demonstrated to be present in earthworm castings^13^,^14^,^15^.

Soil influenced by roots, namely the rhizosphere, is considered an environment of complex biological interactions, where many different species soil microorganisms can grow using the large amount of organic compounds released by roots^16^. Rhizosphere microorganisms play important roles in plant physiology. They can facilitate the uptake of many important nutrients such as nitrogen, phosphorus and iron^16^,^17^ and also synthetize complex compounds known to participate in plant growth regulation processes^17^. Most of the microbes growing in the rhizosphere are organothrophs^16^. Therefore, rhizosphere microbes are likely to be positively influenced by organic compounds egested by earthworms.

Compared to the pre-ingested soil, gut contents can contain more concentrated levels of ammonium, amino acids and fatty acids. Further, compounds such as glucose, maltose, formate, acetate, lactate and succinate, which normally cannot be detected in soils, are found in the alimentary canal in large amounts^18^. Additionally, the *in situ* conditions of earthworm guts are likely to favor denitrification. N_2_O and N_2_ emissions from earthworms and denitrification genes were reported to be enriched in the alimentary canal of earthworms^19^. Likewise, the products of metabolic processes happening in the gut can be released in the soil^20^,^21^,^22^,^23^ and benefit microbial communities living even beyond the drilosphere^24^. However, little is known of the effects of earthworms on microbial functions in the rhizosphere.

Sugarcane is one of the most efficient plants in converting sun energy into sugars. Besides that, this plant has also a remarkable need for accumulating silicon (Si), absorbing it more than any other mineral nutrient^25^. Si has been proposed as an essential element for sugarcane, being needed to support cell growth and protect against water loss, pathogens and heavy metal tocixity^25^. The production of sugarcane is of great importance for developing countries, especially Brazil, where it occupies more than 10 million hectares. Sugarcane cropland receives huge amounts of fertilizers and pesticides annually^26^. Elucidating soil processes and the mechanisms by which earthworms can improve biomass production and plant health is of great concern in order to develop more sustainable uses of natural resources in agroecoystems.

Therefore, we hypothesized that microbial functions in sugarcane rhizosphere are altered by earthworms and that functional changes are associated with plant beneficial functions. Hence, we investigated soil microbial functions in response to the presence of *Pontoscolex corethrurus*, a peregrine earthworm species commonly found in sugarcane fields^27^ and throughout the tropics and sub-tropics^28^, in pots growing sugarcane seedlings. As earthworms are known to emit N_2_O as a consequence of denitrification happening in their gut, and as some soil microbial communities have the potential to be a sink for N_2_O^29^ by reducing it to N_2_ through the nitrous oxide reductase enzyme, we also monitored N_2_O concentrations belowground throughout the experiment and determined the abundance of the nitrous oxide reductase gene (*nos*Z) in bulk soil and rhizospheric community at the end of the experiment. CO_2_ concentrations belowground were also reported as an indicative parameter of respiration. Soil used to fill the pots was collected from an experimental farm, sieved and homogenized. Advanced methods of molecular biology for metagenomic whole community shotgun sequencing were performed to reveal the functional profile of the soil microbial community. Appropriate statistical tests were chosen based on homogeneity of variance and normal distribution of the tested variables.

## Results

### Effect of earthworms on plant biomass and soil chemical parameters

**Figure 1:**
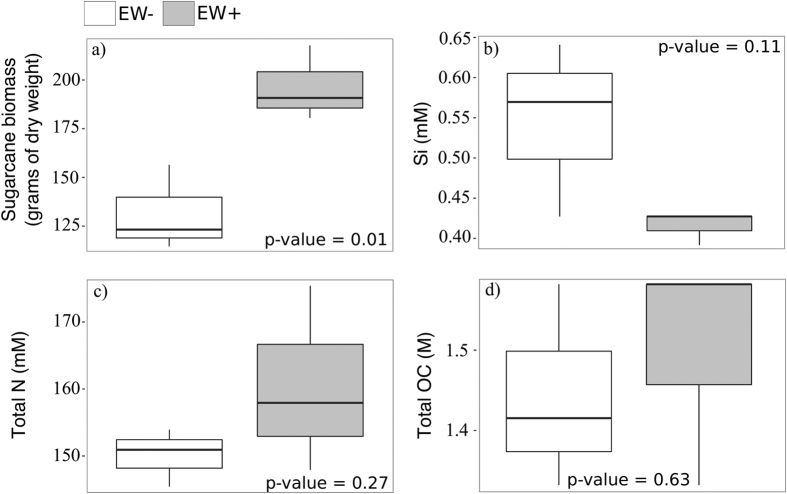
Plant and soil parameters determined after 217 days of greenhouse experiment. Panel (a) indicates plant total biomass (Levene’s test, F > 0.05; Shapiro-Wilk’s test, p > 0.05; t-test, p-value = 0.01). Panel (b) indicates levels of silicon (Si) determined in bulk soil soil samples at the end of the experiment (Levene’s test, F > 0.05; Shapiro-Wilk’s test, p > 0.05; t-test, p-value = 0.11). Panel (c) indicates levels of total soil nitrogen (N) determined at the end of the experiment (Levene’s test, F > 0.05; Shapiro-Wilk’s test, p > 0.05; t-test, p-value = 0.28). Panel (c) indicates the levels of total soil organic carbon (OC) determined at the end of the experiment (Levene’s test, F > 0.05; Shapiro-Wilk’s test, p < 0.05; Kruskal-Wallis, p-value = 0.63). Empty boxes represent the values obtained in the pots without earthworms (EW−) and filled boxes represent the values obtained in the pots with earthworms (EW+).

Data collected at the end of the experiment revealed that mean plant dry mass was significantly higher in the pots with earthworms (EW+) (t-test, p-value = 0.018) (Fig. 1a) and the level of Si in soils was considerably lower than in the pots without earthworms (EW−) (t-test, p-value = 0.11) (Fig. 1b). No significant differences were observed for the levels of total nitrogen (N) (t-test, p-value = 0.29) (Fig. 1c) and organic carbon (OC) in soils (t-test, p-value = 0.63) (Fig. 1d). The detailed results of other chemical parameters of soil are included in the Supplementary (Supplementary Table S1).

### Earthworm populations

At the end of the experiment a mean of 92 individuals (±28.71) of *P. corethrurus* were counted per pot and several cocoons were observed in the three pots. The increase in the number of individuals per pot from the beginning to the end of the experiment was 72 ± 28.71. The mean of the earthworm total biomass (sum of individuals weight) at the end of the experiment was 9.43 (±5.14) grams (g) of fresh weight per pot, almost the same as inoculated (9 g ± 0.57). However, the average weight of the individuals (grams per worm) was considerably lower compared to the initial. The average weight of the individuals inoculated in the pots was 0.45 g (±0.18) and the average weight of the individuals recovered from the pots was 0.10 g (±0.13). This indicates that the experimental conditions favored worm development and reproduction.

### N_2_O and CO_2_ production belowground

**Figure 2:**
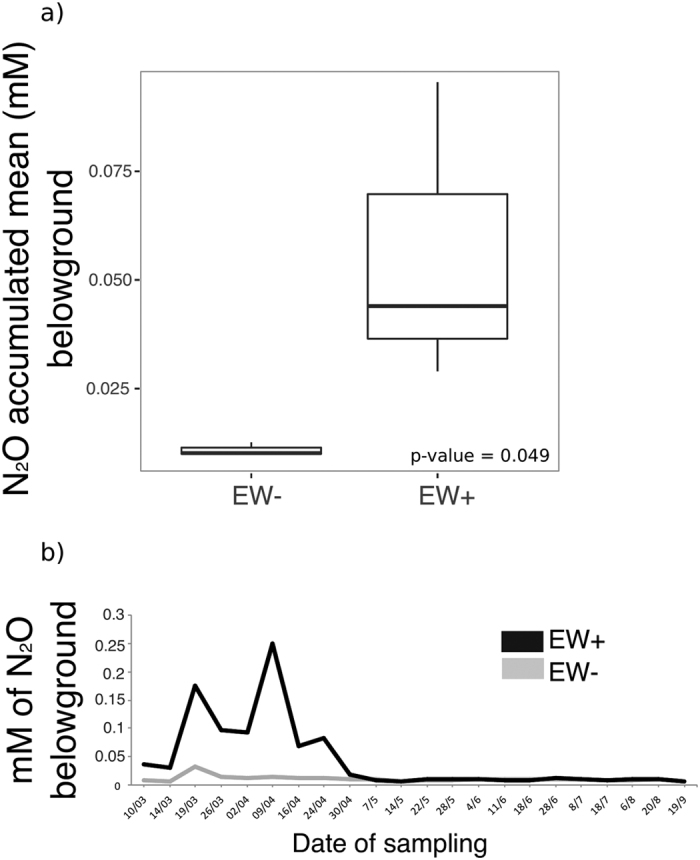
N_2_O concentration belowground (15 cm depth) monitored along the experiment. Panel (a) indicates the accumulated mean of N_2_O concentrations in pots with earthworm (EW+) and without earthworms (EW−) (Levene’s test, F > 0.05; Shapiro-Wilk’s test, p < 0.05; Kruskal-Wallis, p-value = 0.04). Panel (b) indicates 22 values (x-axis) of N_2_O means collected along the experiment (217 days) according to the date of sampling. The black line represents the values obtained in the pots with earthworms (EW+), and the gray line represents the values obtained in the pots without earthworms (EW−).

The accumulated mean of N_2_O concentration belowground (i.e., the sum of all the measurements of concentration obtained from an experimental unit divided by the number of samplings) was significantly higher in EW+ than EW− pots (Kruskal-Wallis test, p-value = 0.049) (Fig. 2a). However averages of N_2_O concentration in a timeline series (Fig. 2b) were significantly higher in EW+ than EW− (Kruskal-Wallis test, p-values < 0.05) only at the beginning of the experiment. After the 60^th^ day (starting from date 30/04), the concentration averages decreased until nearly the same levels found in EW− pots and apart from the sample collected at date 22/05 and 18/07, in which N_2_O was significantly higher in EW+ than EW− (Kruskal-Wallis test, p-values < 0.05), all the others showed no significant differences (Kruskal-Wallis test, p-values > 0.05).

**Figure 3:**
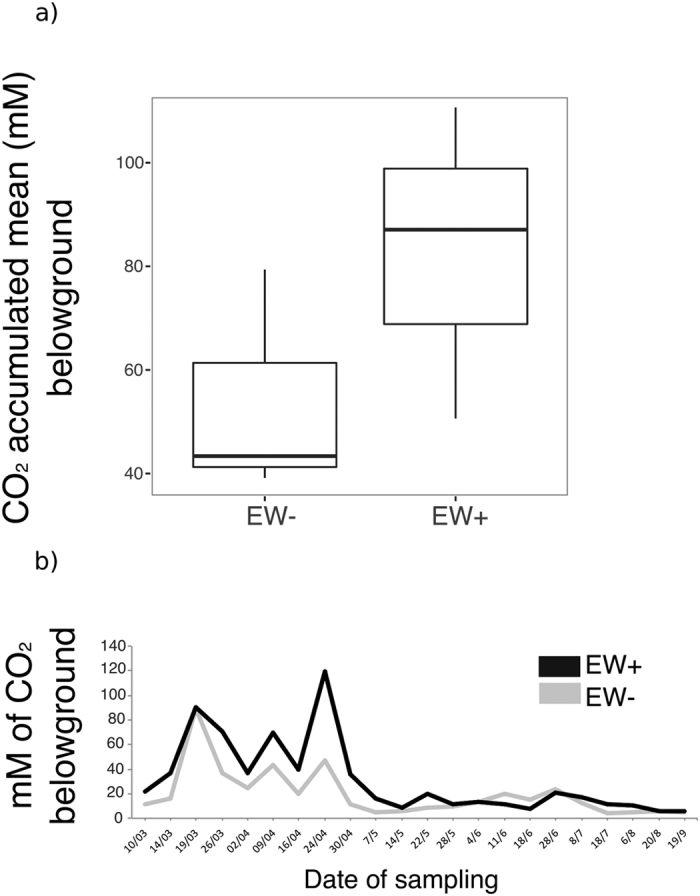
CO_2_ concentration belowground (15 cm depth) monitored along the experiment. Panel (a) indicates the accumulated mean of CO_2_ concentrations in pots with earthworm (EW+) and without earthworms (EW−) (Levene’s test, F > 0.05; Shapiro-Wilk’s test, p > 0.05; t-test, p-value = 0.25). Panel (b) indicates 22 values (x-axis) of CO_2_ means collected along the experiment (217 days) according to the date of sampling. The black line represents the values obtained in the pots with earthworms (EW+), and the gray line represents the values obtained in the pots without earthworms (EW−).

The accumulated mean of CO_2_ concentration belowground was not different in EW+ compared to EW− (t-test, p-value = 0.25) (Fig. 3a). The averages of CO_2_ concentrations in a timeline series (Fig. 3b) were higher in both EW+ and EW− only at the beginning, and started to decrease around day 60^th^. However, CO2 started to decrease a little earlier in EW−, so that CO_2_ concentrations were significantly higher in EW+ for at least 7 days, from date 30/04 until 07/05. Worth noting that the decline period coincided with the decline of N_2_O in EW+.

### Quantification of 16 S rRNA (Bacteria and Archaea) and nitrous oxide reductase gene (nosZ)

**Figure 4:**
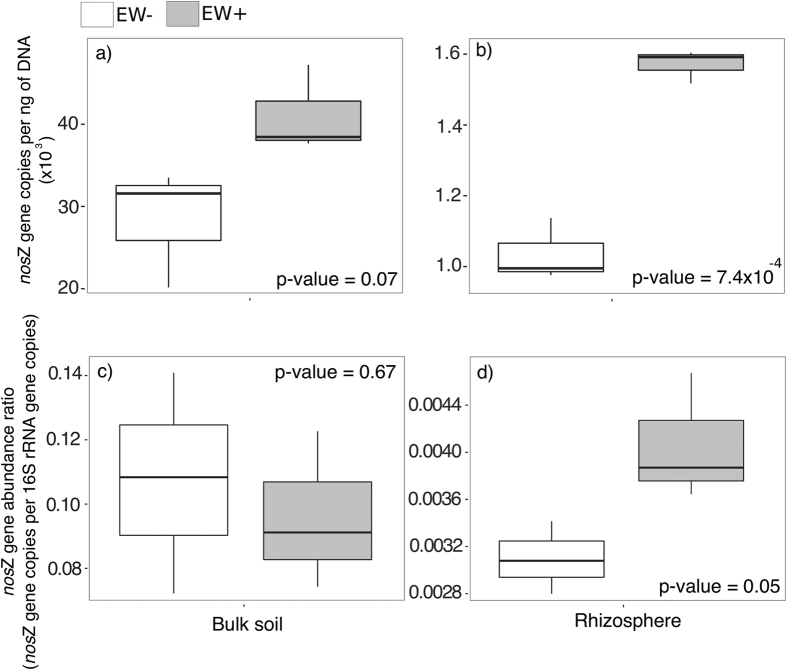
Abundance of nitrous oxide reductase gene (nosZ) determined at the end of the experiment. Panel (a) and (b) indicates the total number of *nos*Z gene copies quantified in bulk soil (Levene’s test, F > 0.05; Shapiro-Wilk’s test, p > 0.05; t-test, p-value = 0.07) and rhizosphere (Levene’s test, F > 0.05; Shapiro-Wilk’s test, p > 0.05; t-test, p-value = 7.4 × 10^−4^), respectively. Panel (c) and (d) indicates the ratio of *nos*Z gene within the prokaryotic community obtained in the bulk soil (Levene’s test, F > 0.05; Shapiro-Wilk’s test, p > 0.05; t-test, p-value = 0.67) and rhizosphere (Levene’s test, F > 0.05; Shapiro-Wilk’s test, p > 0.05; t-test, p-value = 0.05), respectively. The ratio values were obtained by dividing the total abundance of *nos*Z gene copies by the sum of the total abundance of 16 S rRNA genes from Archaea and bacteria. Empty boxes represent the values obtained in the pots without earthworms (EW−) and filled boxes represent the values obtained in the pots with earthworms (EW+).

Bacteria 16 S rRNA gene abundance was enriched significantly in the bulk soil (t-test, p-value = 0.01) from EW+ relative to EW− pots (Table 1). No significant difference was observed for Archaea. The abundance of *nos*Z gene was increased considerably in the bulk soil (t-test, p-value = 0.07) (Fig. 4a) and significantly in the rhizosphere from EW+ compared to EW− (t-test, p-value = 7.4 × 10^−4^) (Fig. 4b). The proportion of *nos*Z for the prokaryotic community (Fig. 4c,d) expressed as the ratio of the abundance of *nos*Z and the 16 S rRNA gene abundances, showed the same tendency (t-test, p-value = 0.05) in rhizosphere of EW+ relative to EW−.

### Metagenomic profiling of microbial functions

**Figure 5:**
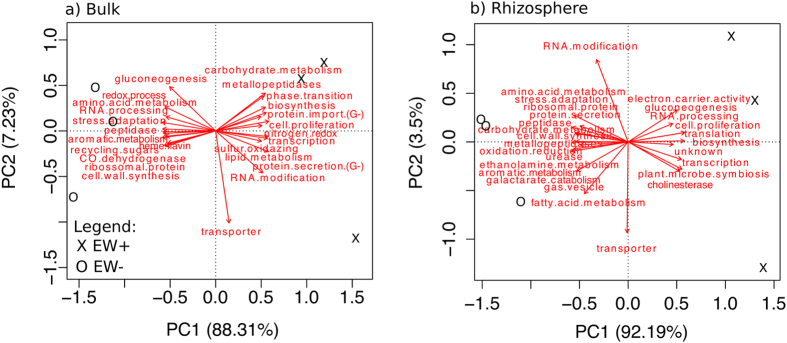
Principal component analysis summarizing the variance of major categories of microbial functions as determined in the metagenomic profiles from bulk soil (**a**) and rhizosphere (**b**) at the end of the experiment. The major categories of functions are composed by more specialized pathways. The complete list of specific pathways of biological importance can be found in Supplementary Fig. S2.

12 metagenomic datasets were obtained (samples from the bulk soil and rhizosphere of the 6 macrocosms). In average, a total of 268,468 ± 149,394 reads passed the quality and length filter per dataset. Analysis of the rarefaction curves revealed good coverage of the diversity of microbial functions (Supplementary Fig. S1). The profiling of metagenomic datasets (total DNA) revealed that earthworm presence significantly changed around 70 microbial functions in both bulk soil and rhizosphere (t-test, p-value < 0.05). For both environments the functions were assigned to major categories based on their descriptions available in the reference database (INTERPRO2GO) or based on current literature when necessary. Figure 5 summarizes the variance of the major categories, in a low-dimensional space using the method of principal component analysis. The entire list of the functions can be found as Supplementary Fig. S2.

Some of the major categories assigned reveal a major pattern. In the EW+ bulk soil, functional genes associated with phase transition, carbohydrate and lipid metabolisms, biosynthesis, translation, protein import/export by Gram-negative (G−) bacteria, redox processes involving sulfur and nitrogen compounds and cell proliferation were enriched relative to EW− bulk soil (Fig. 5a). More specifically, the phase transition major category refers to functions involved in cell motility such as the flagellum (IPR022781, IPR005503) and cell adhesion, referring to a cellular component (pilus) responsible for adhesion (IPR001082). The latter contains phylogenetic signs from G- bacteria. Within the carbohydrate and lipid metabolisms major categories, some functions associated with rapidly metabolisable carbon source (i.e., glucose and fructose) (IPR006256, IPR003755) and catabolism of lipids (e.g., the secretion of lipases) (IPR005152) can be highlighted, respectively. Within the major category of biosynthesis, some of the functions assigned indicate synthesis of complex compounds such as the chaperone protein Skp function (IPR005632), which is involved in the biogenesis of outer membrane proteins^30^. Moreover, although in a very little proportion, genes associated with the production of plant growth regulators were identified (IPR017765).

In the bulk soil from EW−, among others, functions altered were assigned within the major categories of stress adaptation, peptidase activity, and amino acid and aromatic compound metabolisms (Fig. 5a). Additionally, carbon-monoxide dehydrogenase, a function related with a diverse group of facultative chemolitoautotroph bacteria (IPR012780) was enriched in EW− compared to EW+.

In the rhizosphere, worth noting that microbial functions associated with plant-microbe symbiosis, transcription, biosynthesis, transporter and cell proliferation were significantly higher in EW+ compared to EW− (Fig. 5b). More specifically, the functions included within the major category of plant-microbe symbiosis were part of metabolic processes referring to cell host colonization, by microbes known to perform nitrogen fixation (IPR003766)^31^, and to plant growth regulators (IPR005955)^32^,^33^, and to processes mediating cellular interactions within symbiotic interactions (IPR004453)^34^, and to processes participating in secretion systems of protein effectors (IPR007688)^35^. Likewise in bulk soil, metabolic processes involving G- bacteria were also reported in rhizosphere of EW+ (IPR004463). On the other hand, in EW− rhizosphere, among others, the major categories of stress adaptation and peptidase activity were again enriched. Interestingly, EW− conditions presented higher level of genes associated with functions referring to gas vesicle (IPR009430).

### Ecological network interactions of microbial functions

**Figure 6:**
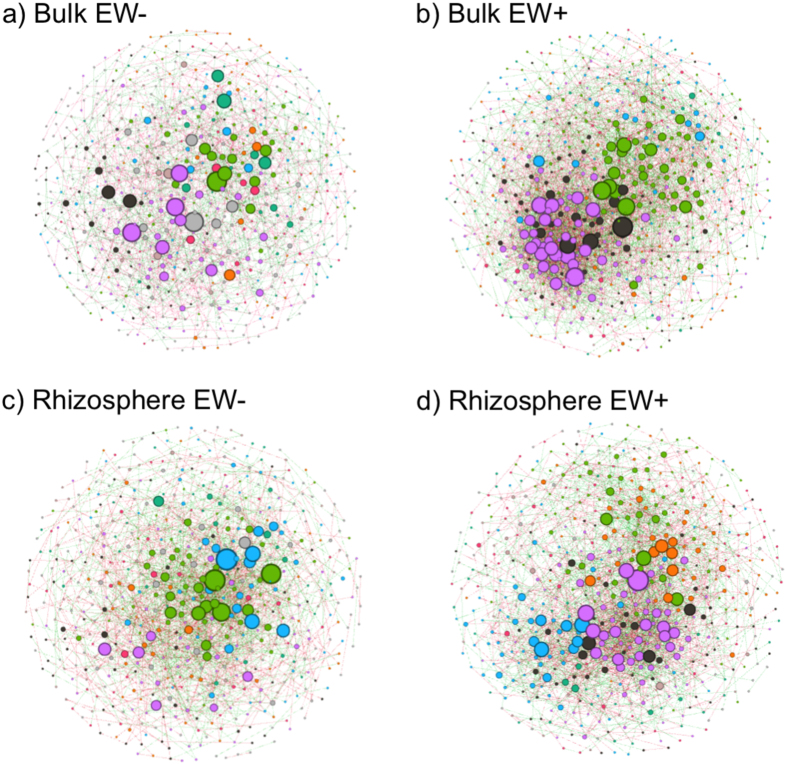
Ecological interactions of microbial functions. Significant (p-value > 0.05) and strong (−0.9> r >0.9) correlations among the most abundant microbial functions. Nodes represent functions and edges represent the correlation between them. Network (a) represents interactions built for bulk EW−, with 642 nodes and 1418 edges (53.88% positive correlations). Network (b) represents interactions built for bulk EW+, with 651 nodes and 3201 edges (52.17% positive correlations). Network (c) represents interactions built for rhizosphere EW−, with 579 nodes and 1737 edges (50.83% positive correlations). Network (d) represents interactions built for rhizosphere EW+, with 564 nodes and 2360 edges (51.91% positive correlations). Different colors indicate different clusters (i.e., modularity), and the nodes were sized according to their importance for the model (i.e., betweenness centrality).

The presence of earthworms in the macrocosms altered ecological interactions among microbial functions, as revealed by the network models (Fig. 6). A decline in the number of clusters (i.e., communities) and an increase in the level of importance (i.e., keystone) of the functions (i.e., nodes) most influencing the models, as indicated by the increase in the values of betweenness centrality of the nodes (Supplementary Table S2), was detected as a major effect of earthworms on microbial communities. Bulk soil of EW− presented 16 clusters while the model built for EW+ bulk soil presented 10 clusters, and 13.97% of the keystone functions in bulk EW+ presented a degree of importance greater than the keystone functions in bulk EW− (Supplementary Table S2). In the EW− rhizosphere, microbial functions were grouped into 20 clusters, and 3.79% of the keystone functions presented greater importance than the keystone function in EW− bulk soil (Supplementary Table S2). In EW+ rhizosphere, microbial functions were grouped into 15 clusters, and 7.26% of the keystone functions presented greater importance than the keystone function in EW− bulk soil (Supplementary Table S2).

## Discussion

Sugarcane biomass was significantly improved in EW+ macrocosms (Fig. 1a). Although not significant, the considerable decrease in Si (Fig. 1b) can be a result of biomass improvement and earthworm-induced microbial activity. Sugarcane is a strong accumulator of Si, and Si fertilization is associated with yield improvements^25^. Further, it has been recently demonstrated that earthworms can improve the Si uptake by plants^36^. These authors^36^ proposed that ingested microbes that can produce exoenzymes in the earthworm gut would be responsible for enhancing the release of Si derived from the degradation of complex organic matter^36^. Our findings suggest that this may also apply to the earthworm-sugarcane system.

The earthworm-induced N_2_O emissions are the consequence of their feeding habits. Experiments have repeatedly demonstrated that N_2_O emissions are associated with microbial processes happening in the gut^19^ and worm-worked soils^37^, where the populations of denitrifiers and dissimilatory nitrate reducers can be more abundant than in bulk soil^18^. Furthermore, the physical process of ingesting microbial cells might kill some of them, releasing N trapped in microbial biomass.

Hence, assuming that earthworms have the constant capacity to increase N_2_O emissions inside the soil, why did these decrease in the present experiment around 60 days after the experiment began? The nitrous oxide reductase, encoded by the gene *nos*Z, is the enzyme that converts N_2_O to N_2_, representing the last step in denitrification^38^. Denitrification is an anaerobic respiratory process in which microbes produce and/or consume N_2_O, representing a biotic source or sink for N_2_O^29^. Therefore, the decrease in N_2_O belowground was a consequence of the increase in *nos*Z gene activity. However, part of the question remains: why after the 60^th^ day? The CO_2_ timeline indicates that soil respiration also declined around 60 days after the beginning of the experiment in both EW+ and EW− conditions (Fig. 3b). Further, except for the dates 30/4 and 07/05 (around 60^th^ and 67^th^ days), no significant differences were detected between the CO_2_ means, which indicates that decrease in CO_2_ was an event independent of the influence of earthworms. Assembling the pots with sieved soil caused extra aeration between the soil particles. Oxygen plays an important role in enhancing CO_2_ emissions from soils by aerobic metabolism belowground^39^,^40^. Thus, these three findings, namely i) the increase in *nos*Z, ii) the decrease in N_2_O, and iii) decrease in CO_2_, suggest that the accumulated earthworm-induced N_2_O was respired by N_2_O reducers mainly after day 60 because the conditions before that could be favoring aerobic respiration due to soil aeration during macrocosm assembling.

Our dataset also suggests that G- bacteria were favored in bulk soil and in rhizosphere of EW+. This agrees with previous findings, suggesting that G- bacteria may have a better ability to survive gut passage than gram-positive (G+) bacteria^24^,^41^,^42^. Or, as an alternative mechanism, previous findings demonstrated that G− population predominates in rhizosphere while G+ predominates in bulk soil^43^. Rhizosphere is known to be dominated by r-strategists while bulk soil is dominated by k-strategists^44^. Therefore, survival from the gut passage, if possible, may not be the only mechanism by which G− can be more positively affected by earthworms. As r-strategists, they could colonize first and grow faster than G+. Bacterial 16 S rRNA gene was significantly increased in bulk soil from EW+ (Table 1). We have found evidences of functional pathways associated with cell motility from G- in bulk soil (IPR001082), which could be due to the need for moving towards the soil zone where earthworms have released nutrients. New experiments need to be performed in order to test this hypothesis.

Earthworm-worked soils can contain large amounts of nutrients concentrated (i.e., ammonium, and sugars) or even nutrients generated exclusively by metabolic processes in their gut (i.e., fermentation)^45^ such as formate, acetate, succinate and lactate^18^. Those easily available organic compounds released in the soil by earthworms may positively affect the metabolism of soil microbes. This may explain why biosynthetic processes were more enriched in EW+ (Fig. 5a,b; Supplementary Fig. S2a, Supplementary Fig. Sb) and cell proliferation functions were observed (Fig. 5a,b; Supplementary Fig. S2a. Supplementary Fig. S2b). Microbes in EW− were lacking this additional source of nutrient. Some of the functions assigned to the major category of stress adaptation response may indicate that microbes in EW− were thriving under relative poor conditions. Mechanisms associated to DNA repair (IPR003717, IPR004504, IPR003180, IPR001631, IPR013765), cytoprotection against diverse environmental stresses (IPR004129)^46^, disturbance in organismal homeostasis (IPR001404), adaptation to nutrient limiting conditions (IPR026253)^47^, activation of minimal catalytic activity under growth-limiting conditions (IPR006377)^48^ were all higher in EW− from bulk or rhizosphere compared to EW+. Sugarcane croplands present a high demand for fertilizers in order to reach a satisfactory level of biomass development. For example, in Brazil, 60–100 kg of nitrogen is applied per hectare annually^49^. In the present experiment, no additional source of nutrient (i.e., fertilizers) was applied to the soils. Therefore, considering the intense competition for nutrients between roots and microbes^9^, it is acceptable that growth conditions were relative limiting in EW−.

Rhizosphere microbes in EW+ were able to invest in functions associated with plant symbiosis (Fig. 5b, Supplementary Fig. S2b). For example, uronate isomerase (IPR003766)^50^ gene shares homology with hormogonium-regulating genes. Hormogonia are gliding filaments specialized for dispersal which are associated with cell host colonization. In some organisms, such as cyanobacteria, this is the phase preceding the differentiation to heterocyst and the expression of nitrogenase^50^. This mechanism has been demonstrated to be important for biological fixation of nitrogen in non-legume plants^31^. The uronate isomerase gene can be found in the genome of several rhizobacteria from the genera *Azorhizobium* (KEGG ID: AZC_3342), *Azospirillum* (KEGG ID: AZLd01370), *Mesorhizobium* (KEGG ID: mll4056), *Sinorhizobium* (KEGG ID: SM_b21354) and *Rhizobium* (KEGG ID:NGR_c32910), among others. Another case of plant-microbe symbiosis is the maleylacetoacetate isomerase (IPR005955), which belongs to a glutathione S-transferase family. These enzymes were demonstrated to be directly involved in regulation of plant growth^32^ and their respective genes can be found in plant-growth promoting rhizobacteria from the genera *Pseudomonas*^33^ and others such as *Bradyrhizobium* (KEGG ID: bll0109), *Sinorhizobium* (KEGG ID: SMc03206), and *Rhizobium* (RHE_CH01748). Additionally, the datasets from EW+ rhizosphere also presented higher levels of functions involved in modulate cell-host interactions (IPR004453)^34^, and functions associated with secretion system (type IV) (IPR007688), responsible for transferring t-DNA and effector proteins to plant cells, which can also participate in beneficial interactions^35^. In comparison to EW−, EW+ rhizosphere had lower enrichment of gas vesicle function (IPR009430). This is a subcellular structure known to happen in several phyla of bacteria and Archaea, which may facilitate buoying cells to the oxygenated layers, working strategically under situations of competition for O_2_^51^. The source of O_2_ in rhizosphere are the root cells, which may loose part of the O_2_ which is delivered to them to the surrounding soil^52^,^53^. The decrease in the need for gas vesicle could be an effect connected with the extra supply of N_2_O by earthworm activity and the increase in *nos*Z gene abundance.

The analysis of network interactions (Fig. 6) suggests that the specific changes observed by contrasting EW+ with EW− (Fig. 5, Supplementary Fig. S2) are supported by modifications that earthworm presence caused to the structure of ecological interactions among microbial functions. The low number of clusters in EW+, compared to EW−, demonstrates that EW+ presented lower need for functional diversification^54^. Niche diversification in soil and rhizosphere microbial communities can be a consequence of increased competition for the same resources^55^. So less niche diversification can be reflecting ecological interactions with less competition for resources and therefore an indicative of higher nutrient availability. Furthermore, the increase in the number of important functions (Supplementary Table S2) in EW+ reflects that more functions were controlling the structure of ecological interactions^54^. Together, these patterns are in consistency with changes detected by the functional profiling (Fig. 5, Supplementary Fig. S2), supporting that worms may have contributed with extra resources to microbes.

**Figure 7:**
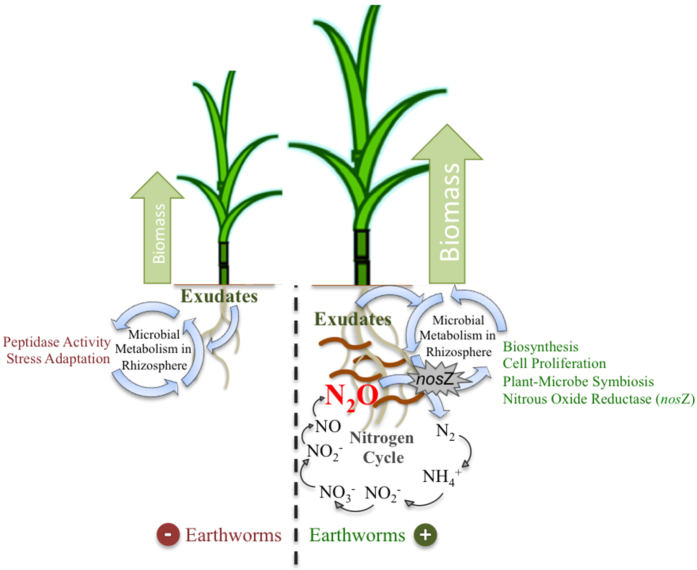
Hypothetical model representing the mechanism by which earthworms may influence rhizosphere microbes in sugarcane. The collective findings in the present study demonstrate that earthworm activity alters microbial functions in the soil (bulk soil and rhizosphere). We propose that the cause for that is the increase in the availability of nutrients and the elevated abundance of N_2_O, both known to be originated during the process of soil digestion inside worm guts, and therefore they may escape from the alimentary canal and be available to the soil microbial communities. Although the complete mechanism might be more complex than here represented, our dataset suggests that these factors may play an important role in enhancing microbial biosynthesis, cell proliferation and plant-microbe symbiosis in the rhizosphere under the influence of earthworms.

Overall, the present study demonstrates that earthworms seem to be important players that positively influence rhizosphere microbes, providing extra resources that may favor them to invest in biosynthetic processes and plant-microbe symbiosis functions. The *nos*Z gene activity was significantly important for microbial community in rhizosphere soils from EW+. We propose, as a hypothetical mechanism, that the production of plant beneficial functions by microbes in the rhizosphere influenced by earthworms may result from the increase in availability of high quality electron donors (i.e., glucose, maltose, formate, acetate, lactate, and succinate)^18^ and the increase in N_2_O as electron acceptor, both products which can escape from the earthworm gut (Fig. 7). The proposed mechanism needs to be tested in further research, in which the influence of the bioturbation process should also be evaluated.

Here we have only measured the abundance of *nos*Z gene clade I (*nos*Z I), however recently, a new clade of this gene (*nos*Z II) has been identified^56^,^57^. There is a possible niche differentiation between these clades. Although both were reported to be present in microbes colonizing roots, *nos*Z I was shown to be significantly more abundant in the rhizosphere^58^. Here we have shown that *nos*Z I is also important for microbial communities in rhizosphere under the influence of the earthworm-induced N_2_O emission. In our dataset, the proportion of *nos*Z I in EW+ bulk was not different from EW−. However, the bacterial population was significantly enriched in EW+ bulk compared to EW−. Therefore, our dataset can support only limited conclusions about the influence of earthworms on nitrous oxide reducers in the bulk soil. Based on recent research^58^, it would be expected for *nos*Z II in bulk soil to show the same response as detected here for *nos*Z I in the rhizosphere. However, further research should address this hypothesis.

The rhizosphere is considered a hotspot for denitrifiers^58^,^59^,^60^, and abundant literature supports that plants can increase N_2_O emission from soils^61^,^62^,^63^,^64^. However, we have performed a microcosm experiment (see Supplementary Methods and Supplementary Fig. S3), using a similar approach (i.e., same plant, same worm and soil from the same origin) and verified that N_2_O production belowground from pots growing sugarcane was not different from the pots without the plant (Supplementary Fig. S3a). In the microcosm experiment, the same effect was observed as was detected in the macrocosm experiment presented here: pots with earthworms showed higher N_2_O emissions belowground. Additionally, the incubation of rhizospheric soils from the pots with and without earthworms showed no significant difference for N_2_O emissions (Supplementary Fig. S3b). While the *in vivo* emissions of N_2_O from *P. corethrurus* (Supplementary Fig. S3c) were significantly higher than the N_2_O emissions of rhizospheric soil from the pots with earthworms (t-test, p-value = 2.5 × 10^−6^). These results reinforce that N_2_O production belowground is dominated by earthworm activity rather than root processes. Additionally, it also reinforces that earthworm-induced N_2_O emission belowground might have little effect on rhizosphere N_2_O respiration in a short-term scale (30 days), as compared with longer time-scale the macrocosm experiment (60 days).

## Methods

### Experimental design

A greenhouse experiment was conducted for 217 days using 100-L plastic pots (41 cm height; 71 cm diameter at the top; 54 cm diameter at the bottom) filled with 70 kg of sieved and homogenized soil (podzolic dark red oxisol; 30% sand, 8% silt and 62% clay), collected from the University of São Paulo - Experimental Station (Piracicaba, Sao Paulo, Brazil), above a 3 cm layer of washed stones. The pots were subjected to natural lighting cycle and natural variation of temperature inside a greenhouse. Piracicaba has a tropical climate, the average of the maximum temperatures along the experiment were around 28.25 °C (±1.38). The soil sieved and homogenized was left resting in pots for 2 weeks until the beginning of the experiment, which was when sugarcane was planted and worms were inoculated. The resting period before beginning the experiment was to stabilize the production of gases resulting from the soil reassembling in pots. Before the beginning of the experiment an airstone (aquarium bubbler, 4 cm height and 1.5 cm diameter) was placed inside the soil, buried in the center of the pot, at 15 cm depth. The airstone was connected with the atmosphere through a silicon tube with a plastic cap that was closed prior to gas sampling. This approach was designed to collect gas samples inside the soil in each one of the pots in order to obtain the concentrations of N_2_O and CO_2_ belowground.

A total of six pots including three replicates with earthworms (EW+) and three without earthworms (EW−) were used to test the influence of earthworms on soil microbiome with growing sugarcane. Soil moisture was monitored with specific sensors (Extech MO750, Nashua, NH, USA) and the humidity was determined at 15 cm depth and maintained at 40% by watering the pots with distilled water when necessary. Plants were obtained from the Sugarcane Center of Technology (CTC). Six seedlings produced by tissue culture, from the same variety (CTC22) and at the same development stage, were planted in each pot. After 90 days, 3 plants from each pot were culled randomly in order to reduce nutrient competition between the remaining plants of the macrocosms. Earthworms (*Pontoscolex corethrurus*) were purchased from Minhobox (Juiz de Fora, MG). Worms were acclimated for 24 hours in extra pots containing the same soil used in the experiment. After this period they were transferred to a plastic container with wet tissue paper and kept for 4 hours for gut “clearance”. Twenty individuals per pot were inoculated in three of the six experimental units just after planting sugarcane seedlings.

Destructive soil and plant sampling was performed at the end of the experiment (217 days). Under field conditions sugarcane is harvested from 12–18 moths after planting. For the specific case of this experiment, the decision was based on the concentrations of GHG in the soil and the size of the plants. Significant differences in N_2_O emissions were observed only during the first 60 days and after 200 days some of the plants were over 2 meters tall, stretching the limits of the greenhouse. Bulk soil was collected (0–10 cm depth) from three equidistant points, considering a 10 cm distance between samples and the position of the silicon tube from the airstone as the centroid. Soil from the different points was homogenized and stored at −80 °C prior to the molecular analysis. The soil samples were subsampled for soil chemical analysis performed at the Soil Analysis Laboratory of University of São Paulo (Department of Soil Science). The three plants in each pot were removed and rhizosphere samples collected by scratching root-attached soil, homogenized and stored at −80 °C prior to molecular analysis. Plant parts (roots and shoots) were oven (60 °C) dried and weighed. Finally, the pots containing earthworms were hand-sieved and all the animals removed, counted and weighed.

### N_2_O and CO_2_ determination

Twenty-two soil atmosphere (belowground) samples were collected per pot from the aeration stones, using syringes periodically along the experiment. The samples were taken all in the morning around 10:00 h, and the time in between the samplings were as follows: the first 16 samplings were taken using an interval of ~7 days, after that, 3 samplings used an interval of ~10 days, and the following 2 samplings used an interval of ~15 days with the last one taken using an interval of ~30 days. N_2_O and CO_2_ were determined using gas chromatography (SRI 8610 C Model, Torrance, CA, USA) configured with the same analytical conditions as described elsewhere^65^ (HayeSep-D and N- packed columns at 81 °C). Average of concentrations was calculated as follows: the values of concentrations measured along the experiment were summed and divided by the number of samplings (Figs 2a and 3a). A timeline plot of the average concentrations for each gas is presented (Figs 2b and 3b).

### Molecular analysis

Total DNA from soil was extracted using the Power Lyzer Soil DNA Isolation Kit (Mo Bio Laboratories Inc., Carlsbad, CA, USA) according to instructions provided by the manufacturer. After extraction, DNA quality was determined in a microliter spectrophotometer (NanoDrop). The quantifications of 16 S rRNA genes from Bacteria, Archaea and *nos*Z (encoding for nitrous oxide reductases) were performed using the StepOnePlus^tm^ Real-Time PCR System (Applied Biosystems, Foster City, CA, USA). The standard curves were obtained from dilutions (10^3^–10^8^ copies of gene per μL) of a known amount of the gene amplified by PCR previously. The reaction mixture included 5 μL of SYBR green 2x reaction mix (Fermentas, Thermo Scientific, Wilmington, DE, USA), 1 μL of each primer (5 μL), 2 μL of ultrapure water and 1 μL of template DNA. The conditions for amplification of the genes 16 S rRNA from Bacteria, 16 S rRNA from Archaea, and *nos*Z were performed as described by Heuer *et al*.^66^, Yu *et al*.^67^ and Henry *et al*.^38^, respectively. Analysis of melting curve of amplicons was performed to confirm the specificity of amplification. After quantification the results were analyzed using the StepOnePlus^tm^ Real Time software v.2.2 (Applied Biosystems, Foster, CA, USA).

Shotgun sequencing of total DNA libraries was performed with Nextera kit according to the manufacturer instructions for the MiSeq reagent kit v2 (500 cycles; Illumina, San Diego, CA, USA). The quality and quantity of DNA used in the kit reactions were determined using spectrophotometer (NanoDrop ND-2000; Thermo Scientific, Wilmington, DE, USA) and fluorometric measurement with the Qubit dsDNA BR assay kit (Moleculas Orobes Life Technologies, Foster, CA, USA). The quantification of DNA in the libraries prior to the last dilution before sequencing, as determined by the manufacturer, was performed using KAPA SYBRFAST qPCR. Libraries were sequenced using an in-house MiSeq Personal Sequencing System (Illumina, San Diego, CA, USA). The metagenomic datasets raw reads are available via MG-RAST under the project name “Metagenomics of sugarcane soils”, via the link “ http://metagenomics.anl.gov/linkin.cgi?project=19145” (files 1–3 refer to the EW− samples, files 4–6 refer to the EW+ samples, letters “b” and “r” indicate whether the reads are from bulk soil or rhizosphere, respectively).

### Statistical analysis

A multivariate analysis was performed for the variables measured using metagenomic approach, for all the others a univariate analysis was performed. In both cases, homogeneity of variance and normality were tested in order to define the most appropriate statistical test to be used in order to detect the significant differences between EW+ and EW−. The significance level (alpha) considered for all the tests was 0.05. For the univariate analysis, to test the null hypothesis of homogeneity and normal distribution the tests, Levene^68^ and Shapiro-Willk^69^ were used. For alpha <0.05 in any of the tests, Kruskal-Wallis^69^ test was implemented, otherwise t-test was implemented^70^. For the multivariate analysis, the homogeneity of variances was tested using Marti Anderson’s (PERMDISP2) procedure, a multivariate procedure analogue to the Levene’s test^71^. Respectively, in bulk soil and rhizosphere, a total of 2,243 and 2,043 variables were assigned as functions encountered in the metagenomic datasets. Hence, the hypothesis of normal distribution was tested based on skewness (Mardia’s test) univariatedly. Only 12% and 15% of the variables, respectively from bulk soil and rhizosphere datasets, were found to be nearly asymmetric as their skew values were found to be two times greater than the standard error of the skewness^72^,^73^,^74^. However, none of the skew values of the variables were above the critical threshold^75^, therefore the datasets were considered to fall within the hypothesis of normal distribution. The analysis of the metagenomic datasets was performed according to the best practices as determined by the Statistical Analysis of Metagenomic Profiles (STAMP) methods, using the effect size and the confidence intervals for assessing biological importance^76^. The t-test (two-sided) was selected using t-test inverted as the method to calculate the confidence intervals of the effect sizes. The effect size is the difference in proportion (DP) of sequences assigned to a given feature in two samples, and it was calculated as follows: DP =  *p*_1_ − *p*_2_. Where *p*_1_ and *p*_2_ are the number of sequences in the two samples assigned to the features of interest (*x*_1_ and *x*_2_) divided by the total number of sequences in the profile (*C*_1_ and *C*_2_) (i.e., *p*_1_ = *x*_1_/*C*_1_; *p*_2_ = *x*_2_/*C*_2_). Error bar plots indicating the p-value with the effect size and associated confidence interval for each function detected to be of significance biological relevance (t-test, p-value < 0.05) were generated (Supplementary Fig. S2).

### Computational analysis

Using PEAR^77^, metagenomic datasets were merged (R1 and R2) and the leftover (not merged) reads from R1 included within the output. Sequences below 50 nucleotides length and Q20 were removed. The screening of the datasets was performed using MEGAN6^78^ by providing the alignments resulting from DIAMOND^79^ against an NCBI-NR database (Feb/2016). The read counts were normalized to the smallest number of reads^78^. Functional profiling was investigated using the INTERPRO2GO database^80^, resulting matrixes were exported using STAMP format for the statistical analysis as described above.

The correlations between the most abundant microbial functions (i.e., all those with abundance grater than the average abundance) were built according to the technique for inferring the sparse correlations for compositional data (SparCC)^81^. This method uses a permutation-based (n = 100) approach to calculate p-values for the interactions, so that only significant (p-values < 0.05) and strong (−0.9> r >0.9) correlations were maintained in the network graph. The graph was visualized with software GEPHI^82^ using the Fruchterman Reingold algorithm. The degree of importance of the nodes was determined by the value of betweenness centrality and the clusters were determined by the modularity of the network, both measures were extracted from GEPHI.

## Additional Information

**How to cite this article**: Braga, L. P. P. *et al*. Disentangling the influence of earthworms in sugarcane rhizosphere. *Sci. Rep.* **6**, 38923; doi: 10.1038/srep38923 (2016).

**Publisher's note:** Springer Nature remains neutral with regard to jurisdictional claims in published maps and institutional affiliations.

## Supplementary Material

Supplementary Information

## Figures and Tables

**Table 1:** Means and standard error for the values of Bacteria and Archaea abundances comparing the treatments with earthworms (EW+) and without earthworms (EW−).

	EW+		EW−	
*Bacteria* (× *10*^*5*^)				
Bulk soil	**4.3**	±0.6	2.6	±0.2
Rhizosphere	3.8	±0.4	3.3	±0.1
*Archaea* (× *10*^*2*^)				
Bulk soil	7.3	±4.6	5	±2.2
Rhizosphere	6.2	±0.7	7.6	±1.6

Values from qPCR were normalized according to the DNA concentration (ng/μl) measured in each sample after extracted from soil. Significant differences between treatments (EW+ and EW−) are represented in bold (t-test, p-value < 0.05).
