# Supplementary material for: Disentangling the influence of earthworms in sugarcane rhizosphere
Source: Sci Rep. 2016 Dec 15;6:38923. doi: 10.1038/srep38923 (PMC5156904; doi:10.1038/srep38923)
Supplement: Supplementary Information [file srep38923-s1.pdf]

Supplementary Information

Title: Disentangling the influence of earthworms in sugarcane rhizosphere

Authors:

Lucas PP Braga

Caio A Yoshiura

Clovis D Borges

Marcus A Horn

George G Brown

Harold L Drake

Siu M Tsai

Supplementary figures, tables and text;

Supplementary Table S1 Soil chemical parameters determined in the bulk soil (217th day)

Supplementary Figure S1 Rarefaction curves obtained from the functional profiling of the metagenomic datasets in bulk (a) and rhizosphere (b).

Supplementary Figure S2 Microbial functions altered in bulk (a) and in rhizosphere (b).

Supplementary Table S2 Values of betweenness centrality* obtained for the first 20 keystone functions found in each network model with (EW+) and without earthworms (EW-)

Supplementary Methods

Supplementary Results

Supplementary Figure S3 Verification study: N2O and CO2 emissions belowground, from rhizospheric soils, and earthworms.

Table S1 | Soil chemical parameters determined in the bulk soil (217th day)

	EW-		EW+	
B	0.4	±0.1	0.3	±0
Cu	0.6	0	0.6	±0.1
Fe	19.3	±0.6	18.3	±1.5
Mn	10.6	±1.7	10.2	±1.1
Zn	8	±6.3	4.6	±1.5
Na	116.3	±19.5	113.7	±13.1
P	10.3	±0.6	9.3	±0.6
S-SO4	40	±33	23	±1.7
K	5.8	±2	3.6	±1.4
Ca	60.3	±4.5	58.7	±3.8
Mg	21	±1	22.7	±0.6
Al	<1		<1	
H+Al	20.7	±1.2	19.3	±1.2
SEB	87.1	±5.6	84.9	±5.4
CEC	107.8	±4.6	104.2	±6.1
pH	6.2	±0.2	6.3	±0.1

SEB: sum of exchangeable bases; CEC: cation exchangeable capacity.
The values for K, Ca, Mg, Al, H+Al, SEB and CEC are represented according the following unit: mmolc.dm-3; for all the others except pH, the unit represented is: mg.dm-3, pH unit is CaCl2 0.01 mol/L. All the variables were statistically tested following the methods as described in the main text. No significant differences were detected.

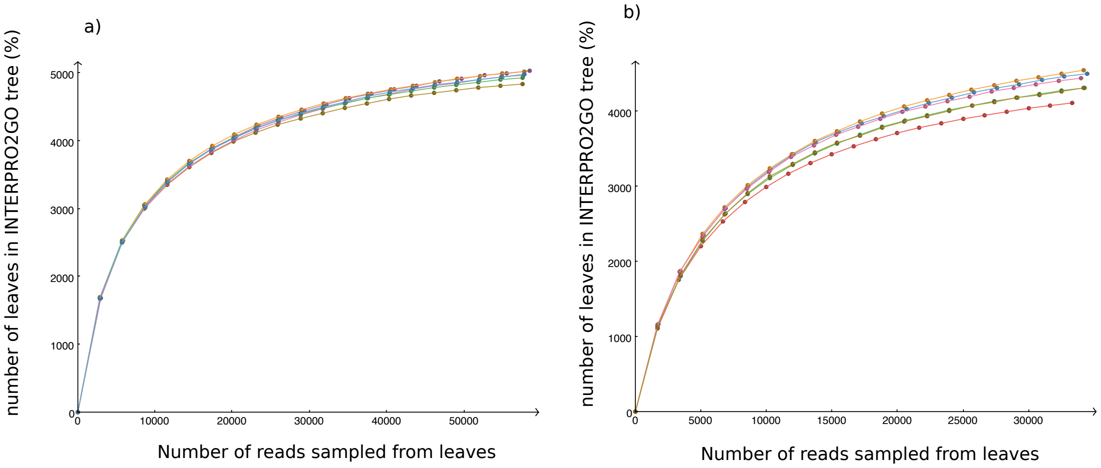

Figure S1 | Rarefaction curves obtained from the functional profiling of the metagenomic datasets in bulk (a) and rhizosphere (b). The curves indicate the coverage of microbial functions assigned to the INTERPRO2GO database for each dataset. The read counts were normalized to the smallest number of reads (MEGAN6). Each leave represents a different function assigned within the tree of functions obtained from MEGAN6. The lines in the plots represent each one of the 12 metagenomic samples obtained at the 217th day after the beginning of the experiment.

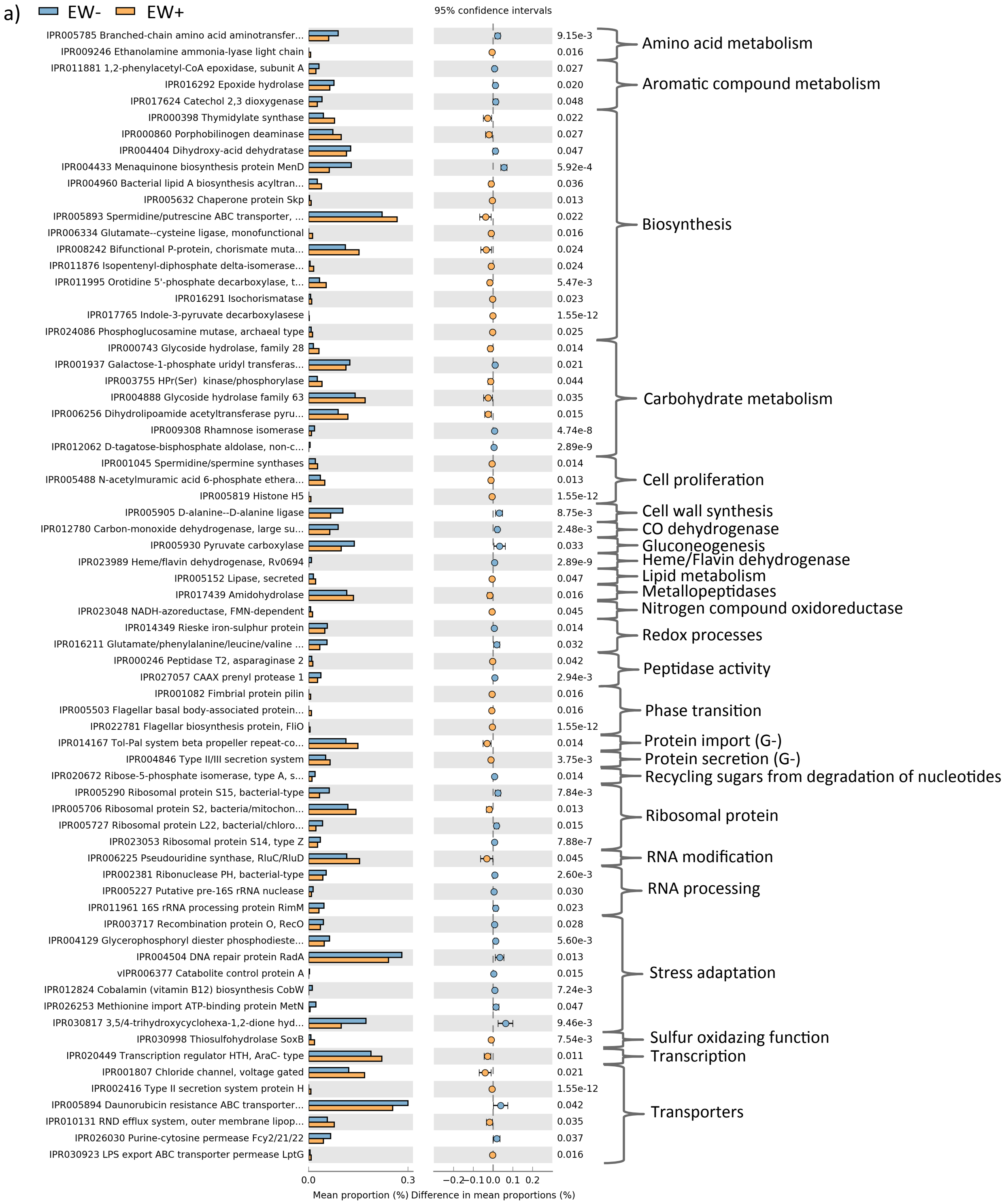
a)
EW-
EW+
95% confidence intervals
IPR005785 Branched-chain amino acid aminotransfer... 9.15e-3
IPR009246 Ethanolamine ammonia-lyase light chain 0.016
IPR011881 1,2-phenylacetyl-CoA epoxidase, subunit A 0.027
IPR016292 Epoxide hydrolase 0.020
IPR017624 Catechol 2,3 dioxygenase 0.048
IPR000398 Thymidylate synthase 0.022
IPR000860 Porphobilinogen deaminase 0.027
IPR004404 Dihydroxy-acid dehydratase 0.047
IPR004433 Menaquinone biosynthesis protein MenD 5.92e-4
IPR004960 Bacterial lipid A biosynthesis acyltran... 0.036
IPR005632 Chaperone protein Skp 0.013
IPR005893 Spermidine/putrescine ABC transporter, ... 0.022
IPR006334 Glutamate--cysteine ligase, monofunctional 0.016
IPR008242 Bifunctional P-protein, chorismate muta... 0.024
IPR011876 Isopentenyl-diphosphate delta-isomerase... 0.024
IPR011995 Orotidine 5'-phosphate decarboxylase, t... 5.47e-3
IPR016291 Isochorismatase 0.023
IPR017765 Indole-3-pyruvate decarboxylase 1.55e-12
IPR024086 Phosphoglucosamine mutase, archaeal type 0.025
IPR000743 Glycoside hydrolase, family 28 0.014
IPR001937 Galactose-1-phosphate uridyl transferas... 0.021
IPR003755 HPr(Ser) kinase/phosphorylase 0.044
IPR004888 Glycoside hydrolase family 63 0.035
IPR006256 Dihydrolipoamide acetyltransferase pyru... 0.015
IPR009308 Rhamnose isomerase 4.74e-8
IPR012062 D-tagatose-bisphosphate aldolase, non-c... 2.89e-9
IPR001045 Spermidine/spermine synthases 0.014
IPR005488 N-acetylmuramic acid 6-phosphate ethera... 0.013
IPR005819 Histone H5 1.55e-12
IPR005905 D-alanine--D-alanine ligase 8.75e-3
IPR012780 Carbon-monoxide dehydrogenase, large su... 2.48e-3
IPR005930 Pyruvate carboxylase 0.033
IPR023989 Heme/flavin dehydrogenase, Rv0694 2.89e-9
IPR005152 Lipase, secreted 0.047
IPR017439 Amidohydrolase 0.016
IPR023048 NADH-azoreductase, FMN-dependent 0.045
IPR014349 Rieske iron-sulphur protein 0.014
IPR016211 Glutamate/phenylalanine/leucine/valine ... 0.032
IPR000246 Peptidase T2, asparaginase 2 0.042
IPR027057 CAAX prenyl protease 1 2.94e-3
IPR001082 Fimbrial protein pilin 0.016
IPR005503 Flagellar basal body-associated protein... 0.016
IPR022781 Flagellar biosynthesis protein, FliO 1.55e-12
IPR014167 Tol-Pal system beta propeller repeat-co... 0.014
IPR004846 Type II/III secretion system 3.75e-3
IPR020672 Ribose-5-phosphate isomerase, type A, s... 0.014
IPR005290 Ribosomal protein S15, bacterial-type 7.84e-3
IPR005706 Ribosomal protein S2, bacteria/mitochon... 0.013
IPR005727 Ribosomal protein L22, bacterial/chloro... 0.015
IPR023053 Ribosomal protein S14, type Z 7.88e-7
IPR006225 Pseudouridine synthase, RluC/RluD 0.045
IPR002381 Ribonuclease PH, bacterial-type 2.60e-3
IPR005227 Putative pre-16S rRNA nuclease 0.030
IPR011961 16S rRNA processing protein RimM 0.023
IPR003717 Recombination protein O, RecO 0.028
IPR004129 Glycerophosphoryl diester phosphodieste... 5.60e-3
IPR004504 DNA repair protein RadA 0.013
vIPR006377 Catabolite control protein A 0.015
IPR012824 Cobalamin (vitamin B12) biosynthesis CobW 7.24e-3
IPR026253 Methionine import ATP-binding protein MetN 0.047
IPR030817 3,5/4-trihydroxycyclohexa-1,2-dione hyd... 9.46e-3
IPR030998 Thiosulfohydrolase SoxB 7.54e-3
IPR020449 Transcription regulator HTH, AraC- type 0.011
IPR001807 Chloride channel, voltage gated 0.021
IPR002416 Type II secretion system protein H 1.55e-12
IPR005894 Daunorubicin resistance ABC transporter... 0.042
IPR010131 RND efflux system, outer membrane lipop... 0.035
IPR026030 Purine-cytosine permease Fcy2/21/22 0.037
IPR030923 LPS export ABC transporter permease LptG 0.016
Amino acid metabolism
Aromatic compound metabolism
Biosynthesis
Carbohydrate metabolism
Cell proliferation
Cell wall synthesis
CO dehydrogenase
Gluconeogenesis
Heme/Flavin dehydrogenase
Lipid metabolism
Metallopeptidases
Nitrogen compound oxidoreductase
Redox processes
Peptidase activity
Phase transition
Protein import (G-)
Protein secretion (G-)
Recycling sugars from degradation of nucleotides
Ribosomal protein
RNA modification
RNA processing
Stress adaptation
Sulfur oxidazing function
Transcription
Transporters
0.0
0.3
−0.2
−0.1
0.0
0.1
0.2
Mean proportion (%)
Difference in mean proportions (%)

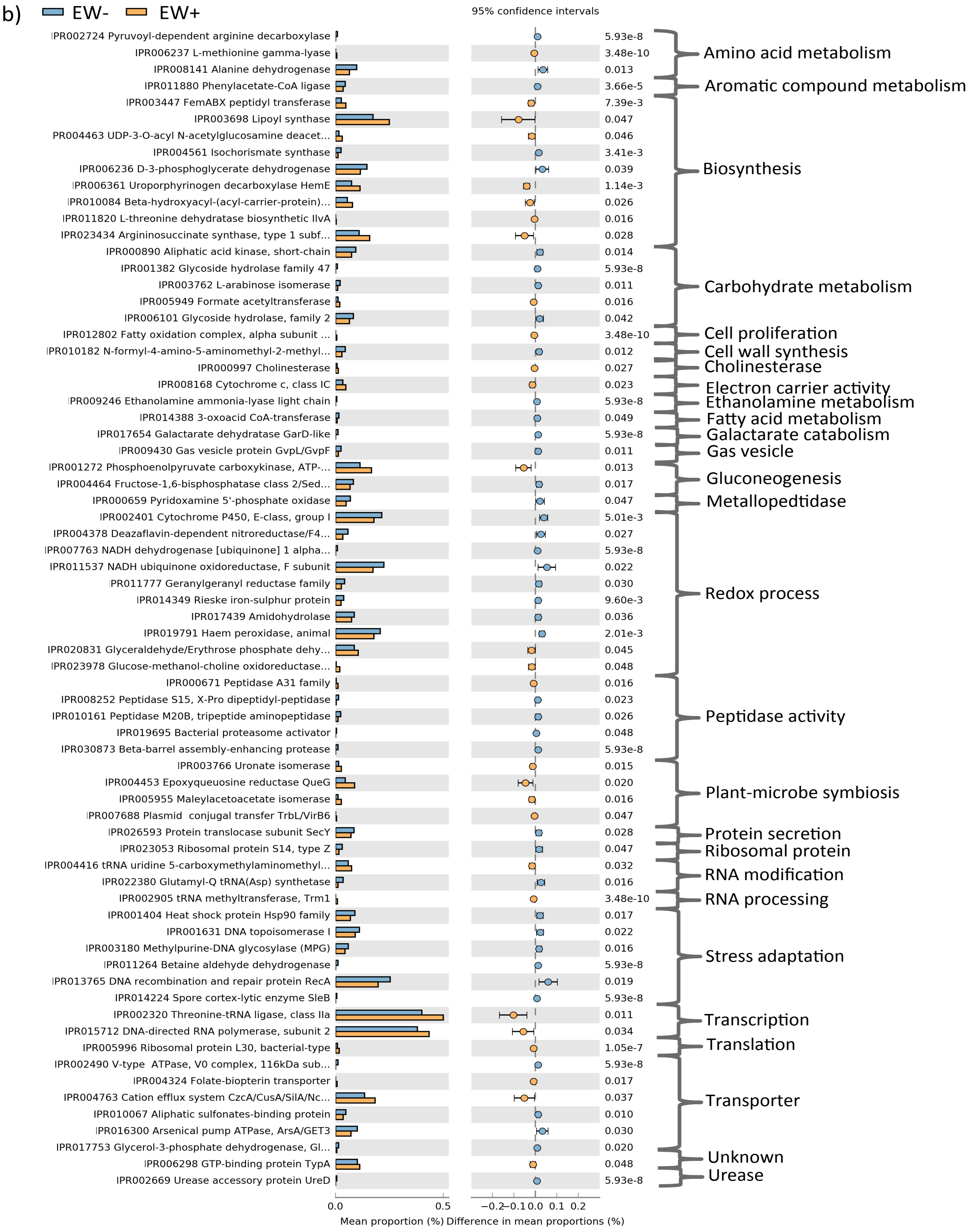

Figure S2 | Microbial functions altered in bulk (a) and in rhizosphere (b). The error bar plots indicates the p-value of the functions with the effect size and associated confidence interval for each function detected to be of significant biological relevance (t-test, p-value<0.05). The color code indicated in the confidence intervals shows if the enrichment was higher in EW- (blue) or in EW+ (orange).

Table S2 | Values of betweenness centrality* obtained for the first 20 keystone functions found in each network model with (EW+) and without earthworms (EW-)

Bulk				Rhizosphere			
EW-		EW+		EW-		EW+	
IPR010067	488.51	IPR003346	2424.90	IPR016407	1293.17	IPR025703	1668.95
IPR001406	482.58	IPR000269	2217.13	IPR005215	1285.72	IPR005372	1255.95
IPR032465	448.96	IPR005771	2157.45	IPR022694	1191.86	IPR000101	1142.79
IPR005746	434.33	IPR004808	2083.24	IPR010228	1089.42	IPR004713	1119.53
IPR004387	412.82	IPR001241	1963.69	IPR004589	898.97	IPR003514	1112.62
IPR005748	336.82	IPR000821	1895.82	IPR004196	855.25	GO000451	1111.28
IPR000748	336.16	IPR002301	1881.19	IPR001062	792.38	IPR026259	1093.10
IPR006200	326.78	IPR002292	1829.40	IPR001238	743.54	IPR006468	1019.87
IPR004809	311.19	IPR011275	1787.26	IPR026019	714.30	IPR002295	955.54
IPR002028	301.71	IPR010226	1727.91	IPR000269	712.73	IPR003568	942.36
IPR011217	284.82	IPR000206	1661.76	IPR017121	650.79	IPR002292	940.66
IPR004506	275.75	IPR004576	1641.33	IPR001701	648.96	IPR011833	876.80
IPR003995	269.25	IPR004670	1632.89	IPR001406	635.34	IPR004791	866.77
IPR005064	267.36	IPR002524	1571.73	IPR002023	630.31	IPR002151	855.63
IPR006443	266.53	IPR015712	1552.21	IPR001719	623.83	IPR002303	847.44
IPR006314	266.17	IPR012394	1516.03	IPR016202	557.10	IPR004807	819.57
IPR003997	260.17	IPR023042	1421.11	IPR008248	545.89	IPR005750	818.25
IPR000522	256.95	IPR023051	1413.71	IPR001088	541.72	IPR000043	816.09
IPR000787	246.50	IPR002320	1411.89	IPR005704	539.29	IPR002139	815.65
IPR019927	233.39	IPR001088	1408.84	IPR004373	534.73	IPR005746	813.39

* Measured according to the number of shortest paths between any two nodes that pass through one particular node. High values indicate high influence of the node on the model.

Supplementary Methods

Microcosm experiment

Plastic pots (5L) were filled with sieved and homogenized soil collected from the same location as was for the macrocosm experiment (University of Sao Paulo – Experimental Station, Piracicaba) and incubated in the greenhouse for 30 days with and without sugarcane seedlings and with and without earthworms (n=4). The earthworms from the same specie (*P. corethrurus*) were obtained from a specialized producer (Minhobox) and followed the same procedure of pre-incubation as described for the macrocosm experiment. Two sugarcane seedlings at the same developmental stage were planted per pot and 20 individuals of young earthworms were inoculated per pot. For sampling the gas belowground it was used the same method described for the macrocosm experiment with airstones buried (15 cm depth) in the center of pots.

After 30 days gas samples belowground were collected and a destructive sampling was performed, and rhizospheric soil and earthworms were incubated for gas measurements. 1 gram (fresh weight) of rhizospheric soil (n=3) from the pots with earthworms (EW+) and without it (EW-), or 1 individual of earthworm (n=3) from the pots with sugarcane (SC+) and without it (SC-) was incubated for 5 minutes inside 10 ml syringes in the dark at room temperature. Prior to the incubation, worms were washed in sterilized water, dried with a paper towel and transferred to the 10 ml syringe. After transferring the rhizospheric soil or the earthworm, the volume of the syringe was set to 10 ml and the plastic cap (replacing the needle) was closed, so that there was no variation in the pressure and the atmosphere inside was not exchanged with the outside. After incubation time the syringe was connected to the chromatograph, the plastic cap of the syringe was opened and a sample of air was injected. After injection of the syringe air into the chromatograph, worms were removed from the syringes and weighted (0.29 g ±0.07). Therefore the gas emission from earthworms was normalized per gram of the individual by dividing the concentrations obtained by the fresh weight of the worm incubated. The variables measured were tested statistically for homogeneity and normal distribution prior to the identification of appropriate statistical tests for detecting differences between the means.

Supplementary Results

Gas emissions from the incubations

The N_2O emissions (Supplementary Figure S3a) detected belowground were different (Kruskal-Wallis, p-value=0.01) among the treatments. N_2O emissions from the pots with only sugarcane (EW-SC+) were not different from the emissions of the pots with only soil (EW-SC-) (Dunn's test. p-value=0.60). However, the N_2O emissions from the pots with earthworms were significantly higher compared to the emissions of the pots without worms (i.e., EW+SC- compared to EW-SC-, Dunn's test, p-value=0.009346; and EW+SC- compared to EW-SC+, Dunn's test, p-value=0.03759; and EW+SC+ compared to EW-SC-, Dunn's test, p-value=0.01157; and EW+SC+ compared to EW-SC+, Dunn's test, p-value=0.04496). Although, the N_2O emissions between the pots with worms were not different (i.e., EW+SC- compared to EW+SC+, Dunn's test, p-value=0.94).

Differences were also detected for the CO_2 emissions (Supplementary Figure S3d) from belowground (Kruskal-Wallis test, p-value=0.008). CO_2 was higher in all the pots with earthworms or with sugarcane (i.e., EW-SC+ compared to EW-SC-, Dunn's test, p-value=0.08; EW+SC- compared to EW-SC-, Dunn's test, p-value=0.04; EW+SC+ compared to EW-SC-, Dunn's test, p-value=0.0006), and no significant difference were detected between them (Dunn's test, p-value>0.05). N_2O and CO_2 emissions of rhizospheres, (Supplementary Figure S3b, e), were not different (t-test, p-value>0.05). *In vivo* emissions of worms (Supplementary Figure S3c, f) were not different for N_2O (t-test, p-value>0.05), but CO_2 emissions of worms from pots with sugarcane were significantly higher compared to the pots without plants (t-test, p-value=0.00929). Further, *in vivo* emissions of N_2O of *P. corethrurus* were significantly higher than the N_2O emissions of rhizospheric soil from the pots with earthworms (t-test, p-value=2.5×10^{-6}).

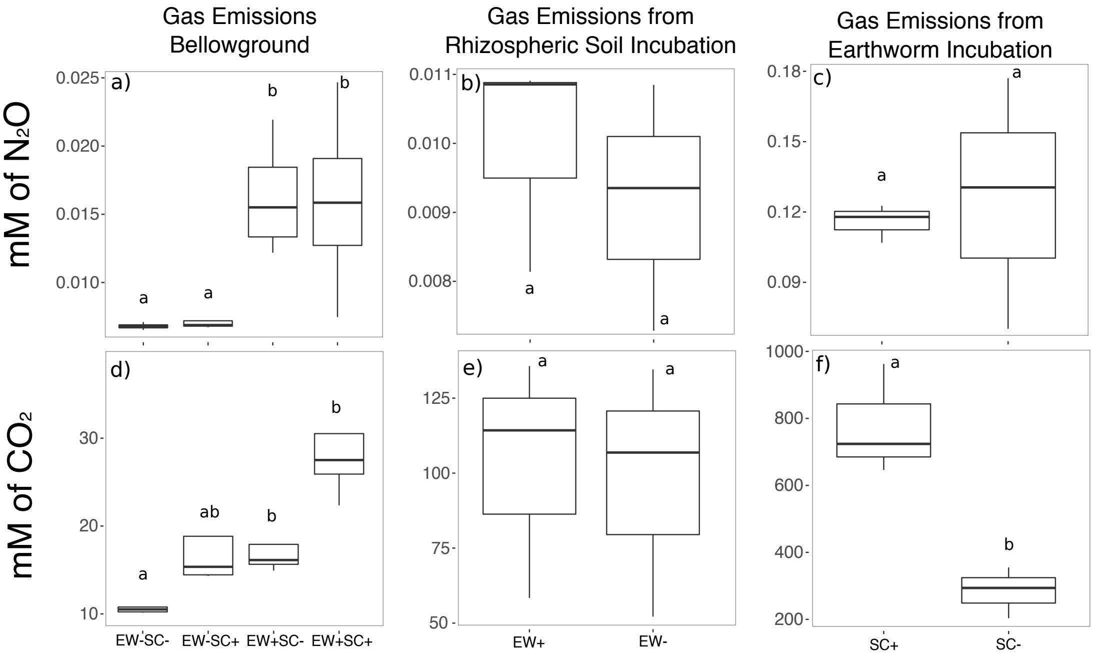

Figure S3 | Verification study: N_2O and CO_2 emissions belowground, from rhizospheric soils, and earthworms. Panels a), b) and c) show N_2O concentrations (mM). Panels d), e) and f) show CO_2 concentrations (mM). Panels a) and d) show the gas concentrations belowground at the end of the experiment (day 30th) for the treatments (n=4): without earthworms and sugarcane (EW-SC-), with sugarcane (EW-SC+), with earthworms (EW+SC-), and with both (EW+SC+). Panels b) and e) show the mean concentration of gas emitted of rhizospheric soils (1 gram of fresh weight) from the pots with earthworms (EW+) and without it (EW-). The panels c) and f) show the *in vivo* gas emissions per gram (fresh weight) of earthworm from the pots with sugarcane (SC+) and without it (SC-). Different letters above the boxes in the panels indicate significant differences (p<0.05).
